# SPR imaging biosensor for determination of laminin-5 as a potential cancer marker in biological material

**DOI:** 10.1007/s00216-016-9621-x

**Published:** 2016-05-21

**Authors:** A. Sankiewicz, L. Romanowicz, P. Laudanski, B. Zelazowska-Rutkowska, B. Puzan, B. Cylwik, E. Gorodkiewicz

**Affiliations:** Department of Electrochemistry, Institute of Chemistry, University of Bialystok, Ciolkowskiego 1K, 15-245 Bialystok, Poland; Department of Medical Biochemistry, Medical University of Bialystok, A. Mickiewicza 2C, 15-089 Bialystok, Poland; Department of Perinatology, Medical University of Bialystok, M. Sklodowskiej-Curie 24A, 15-276 Bialystok, Poland; Department of Pediatric Laboratory Diagnostics, Medical University of Bialystok, Waszyngtona 17, 15-269 Bialystok, Poland

**Keywords:** Laminin-5, Surface plasmon resonance imaging, Biosensors, Cancer marker

## Abstract

A new method for the selective determination of laminin-5 concentration using a biosensor and surface plasmon resonance imaging (SPRI) technique is presented. A biosensor based on the specific interaction of laminin-5 with rabbit polyclonal antibody was constructed. The analytically useful dynamic response range of the biosensor is between 0.014 and 0.1 ng mL^−1^. The detection limit is 4 pg mL^−1^. The potential influence of interferences on the SPRI signal was investigated, and the high selectivity of the biosensor was confirmed. In order to demonstrate the potential application of the biosensor, laminin-5 concentration in blood plasma was determined. The results were compared with the laminin-5 concentration obtained by the commercial enzyme-linked immunosorbent assay (ELISA) kit. A comparison of results from healthy donors obtained by SPRI measurement and ELISA indicates that they are close and shows good agreement with the data reported in the literature. The plasma samples of bladder cancer patients gave higher concentration measured with specific biosensor than by ELISA assay. The study shows the clear difference in concentration of laminin-5 in healthy humans and patients with bladder cancer. Extensive clinical studies using the newly developed method can result in an increase in the use of laminin-5 as a potential cancer marker.

**Electronic supplementary material:**

The online version of this article (doi:10.1007/s00216-016-9621-x) contains supplementary material, which is available to authorized users.

## Introduction

Surface plasmon resonance imaging (SPRI) is a tool to monitor biomolecular interactions. It is a label-free optical detection technique and allows real-time monitoring of reaction between biomolecules. The combination of SPRI technique with the biosensors is at the forefront of analytical methods of multi-analyte measurements [1]. This method measures the changes of refractive index caused by molecules bound to the metal surface. Surface plasmon resonance (SPR) is generated at a thin metal surface when surface plasmons are excited by the light beam at an appropriate incident angle. This angle strongly depends on the refractive index of the dielectric material at the interface. When the analyte binds the ligand on the metal film, the interfacial architecture changes. The SPRI signal is dependent on the change in the wavelength and the angle of polarization of light. It is also directly proportional to the mass change on the surface of the metal [2]. The biosensor is usually gold-coated glass with a layer of active biomolecule as a receptor for a determined compound. They are based on very specific interactions between enzyme and inhibitor or between antigen and antibody. This allows determining the concentration of the analyte directly in the sample [3]. SPR imaging biosensors have been increasingly applied in medical diagnosis in which rapid and sensitive methods are required for the determination of substances (presence and/or amount) that are potential markers of disease and especially cancer markers [4–8].

Recent progress in science has highlighted the importance of extracellular matrix (ECM) in regulation of cellular behavior and major developmental processes during cancer progression. Understanding how ECM composition and its deregulation influence the development and progression of diseases may help in an early cancer diagnosis.

The extracellular matrix is a complex network of molecules secreted by cells. They are divided into four main groups of proteins: collagens, elastins, structural glycoproteins, and proteoglycans. Glycosaminoglycans and other polysaccharides are also involved in its maintenance. The ECM provides structural and biochemical support of the surrounding cells. ECM is involved in signal transduction between cells, maintaining their shape, and also in the processes of migration and cell growth [9, 10]. In particular, the laminins, a family of extracellular glycoproteins, play an important role in ECM functioning. They are a component of the basement membrane (BM), a specialized form of extracellular matrix, in almost all animal tissues. Laminin molecule is a heterotrimer assembled from α, β, and γ chain subunits. It forms a cross shape with one long arm and three short arms. There are five laminin α chains (α1–α5), four laminin β chains (β1–β4), and three laminin γ chains (γ1–γ3), and each chain type represents a different subfamily of laminins. Currently, there are over 18 laminin isoforms [11].

Laminin-5 (Lm-332, 400 kDa) is the isoform that contains alpha-3, beta-3, and gamma-2 chains, and it is expressed predominantly in the BM [12]. The fragments of γ2 chain are specific for the laminin-5 [13]. Many studies have shown the importance of laminin-5 in promoting tumor invasion through interactions with several cell-surface receptors (including α6β4 and α3β1 integrins, epidermal growth factor receptor, and syndecan 1) and other basement membrane components (including type VII collagen) and stimulation of cell migration and/or invasion after BM degradation. Based on its function, it may support cancer cell invasion. Several immunohistochemical studies have shown a positive correlation between laminin-5 expression and tumor invasiveness [14–18].

Each of the laminin-5 constituent may be degraded into smaller fragments. The laminin-5 can function as a motility factor or as an adhesive factor, depending on the state of proteolytic processing. One possibility is that plasmin can cleave the α3 chain. This makes the laminin-5 an adhesive agent. Another possibility is the cleavage of the γ2 chain by some matrix metalloproteases (MT1-MMP, MMP-2) and the triggering cell migration over laminin-5 [19].

The most common methods used for laminin-5 detection and determination are immunohistochemistry and enzyme-linked immunosorbent assay (ELISA), respectively [20]. Especially, expression of the laminin γ2 chain of tumors was analyzed [21–23]. The most common methods used for laminin-5 detection are time consuming, they are not quantitative, and they require the use of the labels. The serum concentration of the N-terminal fragments of the γ2 chain by immunoassay was measured [24].

As long as ELISA is practically the only available method for laminin-5 determination, this method cannot be validated by comparison with another method. This opportunity provides the SPRI method developed in this work, which is label-free and operates with entirely different methods of creation of the analytical signal. Thus, ELISA and newly developed SPRI methods can be used for mutual validation of the results.

The biosensor on the basis of laminin-5 interaction with specific antibody was constructed. Next, the analytical parameters of SPRI biosensor were optimized and the ability of biosensor for determination of laminin-5 in biological samples was checked. Comparison with standard methods, ELISA, and Western blot was performed.

## Materials and methods

Recombinant human protein laminin-5 (*M* = approx. 900 kDa) as standard (Abcam, USA, http://www.abcam.com/), rabbit polyclonal antibody specific to human laminin-5 (*M* = 170 kDa) (Abcam, USA, http://www.abcam.com/), cysteamine hydrochloride, *N*-ethyl-*N*′-(3-dimethylaminopropyl)carbodiimide (EDC), human albumin, glycoprotein GPIIb/IIIa (Sigma, Steinheim, Germany, http://www.sigmaaldrich.com/safc/facilities/steinheim-germany.html), *N*-hydroxysuccinimide (NHS) (Aldrich, Munich, Germany, http://www.sigmaaldrich.com/germany.html), and photopolymer Elpemer SD 2054 and hydrophobic protective paint SD 2368 UV SG-DG (Peters, Kempen, Germany, http://www.peters.de) were used as well as absolute ethanol, acetic acid, hydrochloric acid, sodium hydroxide, sodium chloride, sodium carbonate, sodium phosphate, potassium phosphate, sodium acetate, potassium chloride, magnesium chloride (POCh, Gliwice, Poland, http://www.poch.com.pl/). HBS-ES buffer, pH = 7.4 (0.01 M HEPES, 0.15 M sodium chloride, 0.005 % Tween 20, 3 mM EDTA); acetic buffer, pH = 3.79–5.57; phosphate-buffered saline (PBS), pH = 7.4; phosphate buffer, pH = 7.17–8.04; and carbonate buffer, pH = 8.50–9.86 (BIOMED, Lublin, Poland, http://www.biomed.lublin.pl) were used as received. ELISA kit for laminin-5 determination (USCN Life Science, Inc., People’s Republic of China, www.uscnk.com) was used. Aqueous solutions were prepared with Milli-Q water (Simplicity® Millipore). Argon N 5.0 with an Ar content ≥99.999 % was used (Air Liquide Polska Sp. z o.o., Poland).

### Chip preparation

Gold chips were manufactured as described in a previous paper [6, 25]. The gold surface of the chip was coated with a photopolymer and printed hydrophobic paint as described in the paper [26]. The chip has nine active sites with 12 free gold surfaces each. The constructed system can simultaneously measure the nine different samples. Twelve individual SPRI signals for each sample can be obtained.

### Atomic force microscopy measurements

The atomic force microscopy (AFM) measurements were performed with a commercial Ntegra Prima scanning probe microscope (NT-MDT, Russia) using a tapping mode in ambient conditions. Etalon probes (NT-MDT, Russia) with resonance frequency around 140 kHz and curvature radius less than 10 nm were used.

### Antibody immobilization

The gold chips with the previously prepared photopolymer layers were rinsed with ethanol and water and dried under a stream of nitrogen. They were then immersed in 20 mM of cysteamine ethanolic solutions for at least 2 h. After this time, the chips again were rinsed with ethanol and water and dried under a stream of argon. Antibody solution in a PBS buffer, activated with NHS (250 mM) and EDC (250 mM), was placed on the amine-modified surface and incubated at 37 °C for 1 h [27]. Activation of the antibody was done by introducing the 1:1 mixture of NHS and EDC in a carbonate buffer solution (pH 8.5) into the antibody solution with vigorous stirring for 5 min. The reaction between activated carboxylic acid groups in the antibody and amines on the SPR chip surface led to antibody immobilization by the covalent amide bond. The bond valence is contingent on matching the antigen binding site to an epitope of antibody. The prepared biosensor was ready for the measurement of laminin-5 concentration. The chip was used immediately.

AFM enables the observation of the surface of the biosensor during its preparation. AFM measurements were performed to confirm the creation of subsequent layers. All measurements were done in ambient conditions. (AFM images of the individual layers on the gold chip surface are presented in the electronic supplementary material).

### SPRI measurement general description

**Fig. 1:**
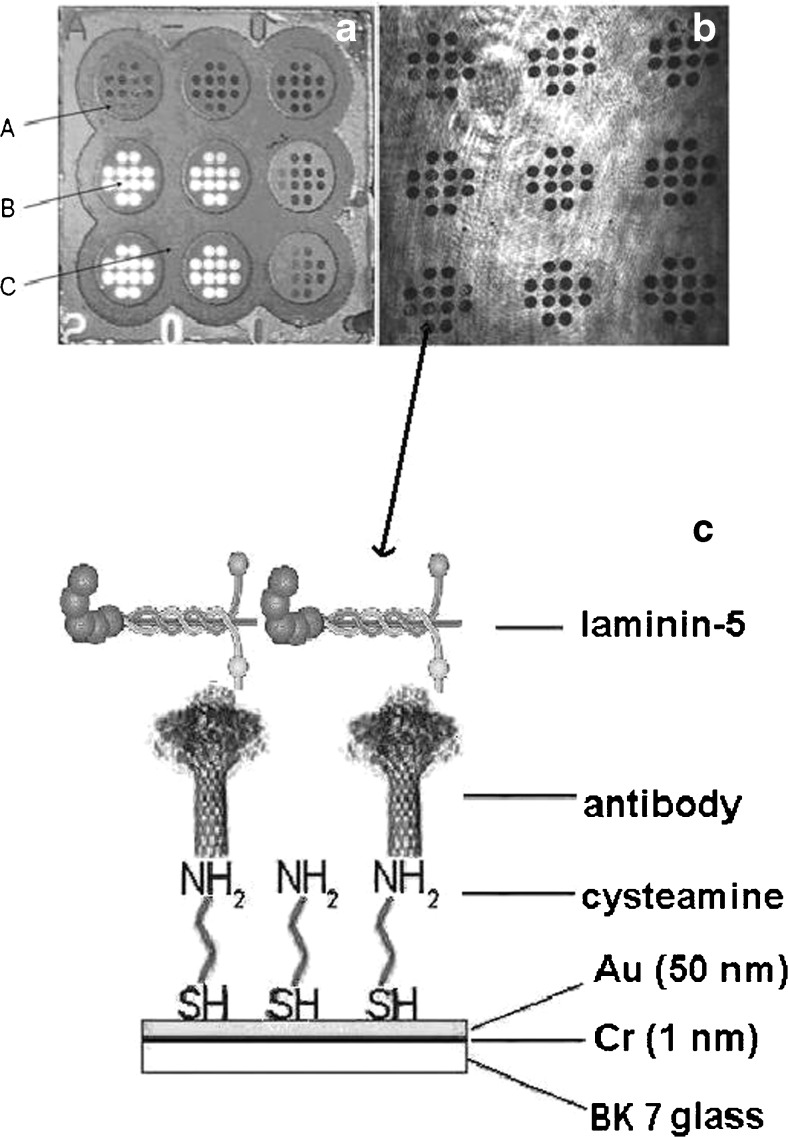
**a** Picture of chip (**A** photopolymer, **B** free gold surface, **C** hydrophobic paint). **b** The SPR image of the chip obtained using a CCD camera. **c** The schematic illustration of the sensor active part

The exact manner of conducting SPRI measurements was described in previous articles [28]. The schematic diagram of the apparatus is given in Ref. [29]. Briefly, the measurements were performed at a fixed angle of incident light and the reflectivity was simultaneously measured across an entire chip surface. The biosensor consists of 9 × 12 spots (Fig. 1a). Measurements were performed across each single spot. Potential differences in the number of pixels on each spot and related measuring error are represented by the precision of measurements. The images were taken after immobilization of the antibody and then after interaction with laminin-5. The contrast values obtained for all pixels across a particular sample single spot were integrated. Thus, the SPRI signal was integrated over the single spot area.

A background correction was applied; i.e., some of the places on the biosensor covered with PBS buffer were used as controls. Non-specific binding was monitored by measuring the signal SPRI in place on the chip without the receptor (ligand). Minimization of the non-specific binding obtained by preparing samples in PBS buffer (NaCl and KCl concentration ∼200 mM) at pH 7.4 near the isoelectric point of the protein. NIH ImageJ version 1.42 software was used to evaluate the SPRI images in 2D form and to convert numerical signal to quantitative signal (AU). The analytical signal, which is proportional to the mass changes caused by binding of laminin, was obtained from subtraction between the signal before and after interaction with laminin-5 for each spot separately. In this manner, 12 individual SPRI signals for each sample were obtained (Fig. 1a).

### Preparation of biological samples

Plasma samples from healthy adult human donors from a Blood Donor Center in Bialystok, Poland, were obtained. Plasma samples from patients with bladder cancer were supplied from the Department of Urology, Regional Hospital, in Bialystok, Poland. Directly after collection, the samples were stored at −20/−70 °C until use.

All the samples were provided after obtaining the consent of the local Bioethical Commission for the study of the biological material collected.

Prepared plasma samples from healthy donors were diluted 1000-fold and those from bladder cancer patients (after preliminary tests) 1000- or 10,000-fold with PBS. Next, 3 μL of the sample dilution was applied to each active site of the biosensor and allowed for 10 min. The volume of the sample was determined by the size of the active place on the chip. The optimum interaction time between antibody and laminin-5 was determined based on the earlier experiments, and it was 10 min. After interaction, the excess solution was removed by rinsing the chip two times with HBS-ES buffer and five times with distilled water. The SPRI measurement was performed. The concentration was evaluated using a laminin-5 calibration curve. Preparation of calibration curve is described in the “Results and discussion” section.

### ELISA measurements

The determination of laminin-5 concentration in the blood plasma was performed using a human laminin-5 enzyme-linked immunoassay kit according to the manufacturer’s instructions. The calibration curve of the ELISA kit is within the range of 15.6 to 1000 ng mL^−1^. The minimum detectable dose of laminin-5 typically is less than 6.1 pg mL^−1^.

### Statistical analysis

All results are given as the mean value ± confidence limits. Student’s *t* test was used for statistical analysis. A *p* value of ≤0.05 was considered significant.

## Results and discussion

### Optimization of antibody concentration

Generally, the usefulness of biosensor is determined by the relevant analytical parameters. The description of analytical capabilities of biosensors includes four sets of parameters: (1) calibration characteristics such as sensitivity, working and linear concentration range, detection, and quantitative determination limits; (2) selectivity and reliability; (3) response time; and (4) reproducibility, stability, and lifetime.

**Fig. 2:**
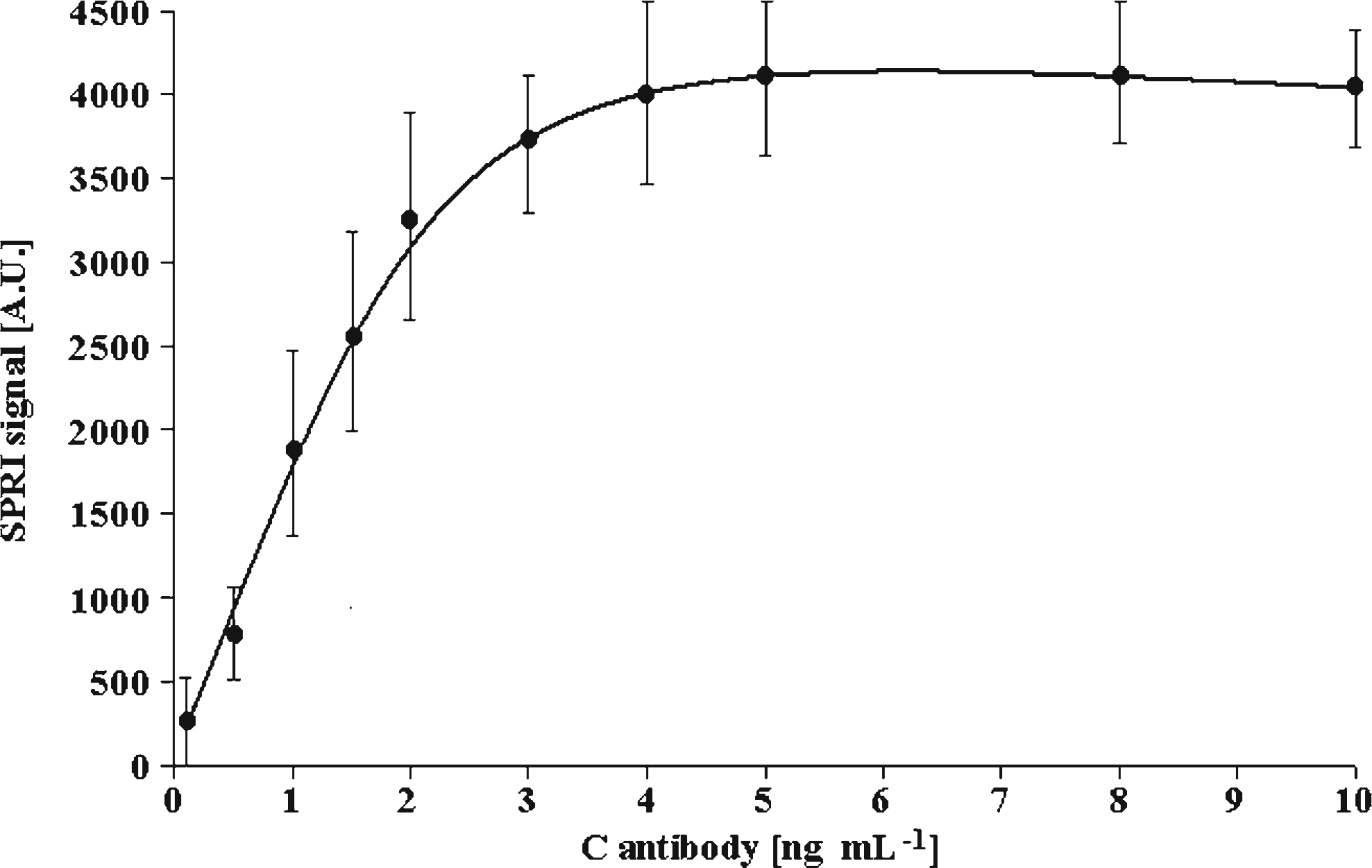
Dependence of SPRI signal (arbitrary units) of antibody-laminin-5 complex on antibody concentration. The laminin-5 concentration is 1 ng mL^−1^. The pH of laminin-5 solution was 7.4. *Error bars* are calculated for 12 independent measurements for each concentration at a 95 % confidence level

The purpose of this investigation was to find the best conditions for determination of laminin-5 concentration. The investigation was performed using the antibody in a concentration range of 0.1–10 ng mL^−1^ at a constant concentration of laminin-5 (1 ng mL^−1^). For this purpose, nine antibody solutions with concentrations 10, 8, 5, 4, 3, 2, 1.5, 1, 0.5, and 0.1 ng mL^−1^ were used. Briefly after chip preparation with specific antibody and laminin-5 binding, the SPRI measurement was performed. The results are given in Fig. 2. The obtained curve is of the Langmuir isotherm type. SPRI signal increased with an increase in antibody concentration from 0.1 to 5.0 ng mL^−1^. The plateau of the signal is observed for antibody concentration above 5.0 ng mL^−1^ (see Fig. 2).

### Influence of solution pH on interaction process

**Fig. 3:**
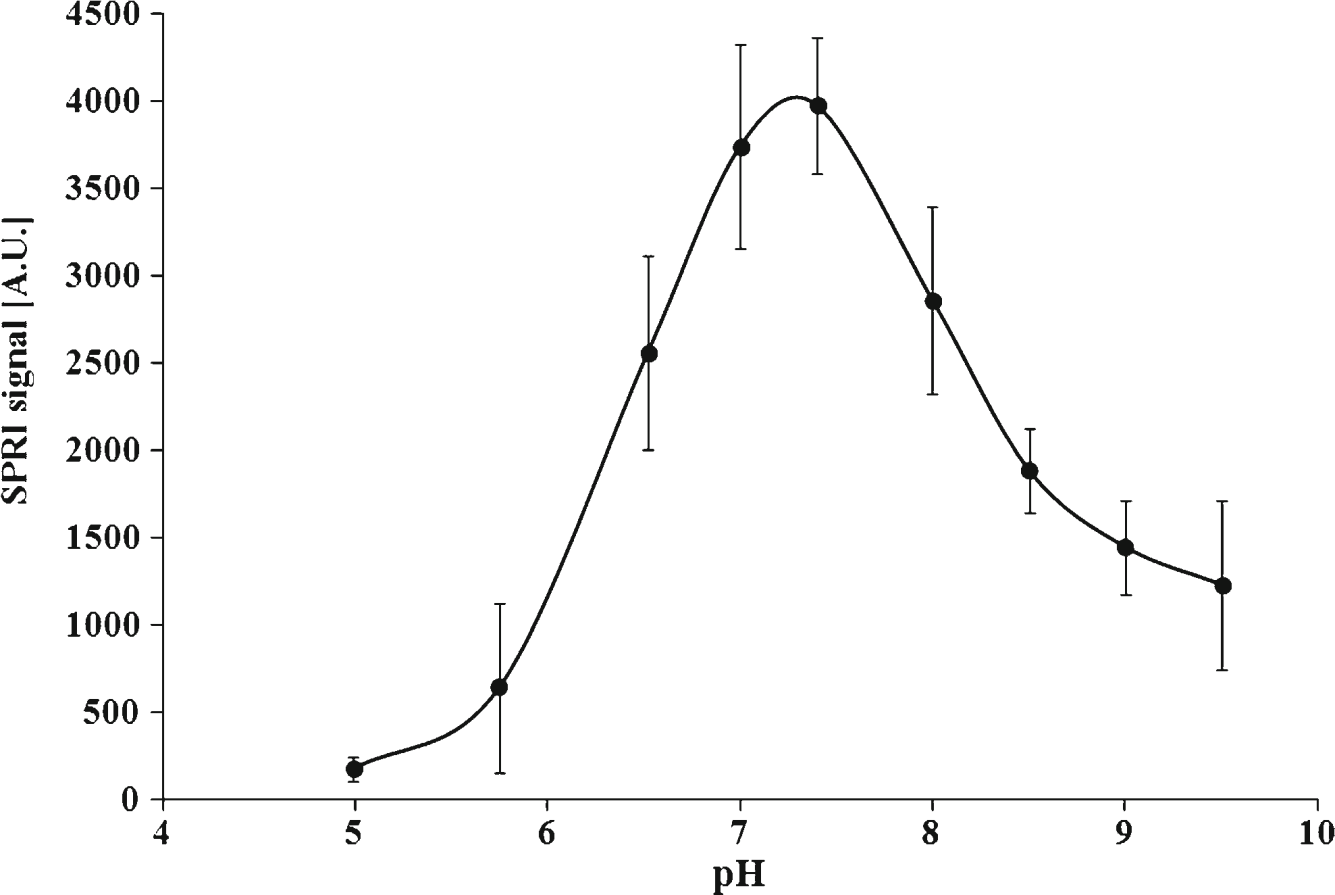
Dependence of SPRI signal (arbitrary units) of antibody-laminin-5 complex on pH. The antibody concentration is 5.0 μg mL^−1^. The laminin-5 concentration is 1.0 ng mL^−1^. *Error bars* are calculated for 12 independent measurements for each antibody solution pH point at a 95 % confidence level

Physiological pH for antigen binding by antibody is 7.4 [30]. The influence of the laminin-5 solution pH on the SPRI signal was studied within a pH range of 4.99–9.50. Biosensors were prepared for measurements at the optimal concentration of antibody (5.0 ng mL^−1^). Experiments were performed at constant laminin-5 concentration (1 ng mL^−1^). The results are shown in Fig. 3. Laminin-5 was bound to use specific antibody at a whole-investigation pH range. The maximum of the SPRI signal was in the pH range of 7.0–7.5. Lower and higher pH values resulted in significantly weaker SPRI signal. The antibody solution pH of 7.4 was selected as an optimal value for further testing.

### Calibration curve

**Fig. 4:**
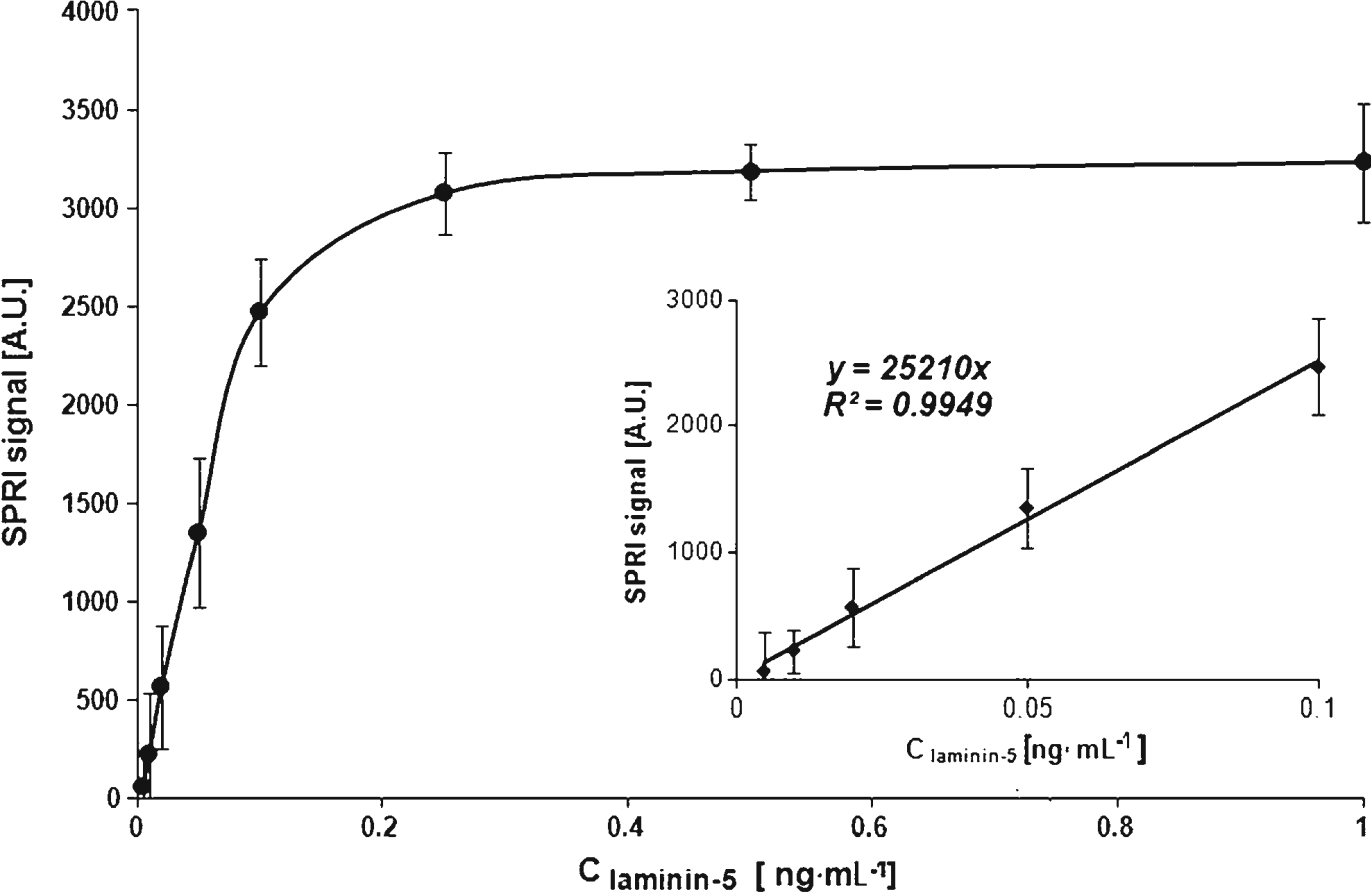
Dependence of the SPRI signal (arbitrary units) on laminin-5 concentration. Linear ranges are on a separate sub-figure. The antibody concentration is 5.0 ng mL^−1^. The pH of laminin-5 solution is 7.4. *Error bars* are calculated for 12 independent measurements for each concentration at a 95 % confidence level

The response of the analytical SPRI signal for laminin-5 was measured within a range of its concentration between 0.005 and 1.0 ng mL^−1^. For this purpose, the chip surface was covered by a monolayer of cysteamine and a layer of immobilized antibody with a concentration of 5 ng mL^−1^. Laminin-5 solutions with different concentrations were put on immobilized antibody for 10 min at pH 7.4. The obtained calibration curve is shown in Fig. 4. That curve represents a Langmuir isotherm type. The calibration of the biosensor gave a linear response to a concentration of laminin-5 in the range of 0.005–0.1 ng mL^−1^. This range is useful for analytical purposes. The plateau of the curve corresponds to saturation of the sensor active points.

The detection limit, calculated on 3 standard deviations (SD) of the blank (PBS buffer) basis [31], is equal to 4 pg mL^−1^. Thus, the limit of quantification (10 SD of the blank) is equal to 14 pg mL^−1^. These values are in an agreement with the lowest results shown in Fig. 4. Thus, linear range between 0.014 and 0.1 ng mL^−1^ can be used for determination of laminin-5 concentration.

### Selectivity of the method

In order to demonstrate the selectivity of the biosensor, the interferences of different compounds to the laminin-5 response were examined. Human collagen type IV, albumin, glycoprotein GPIIb/IIIa, and fibronectin as potential interferents were used. Collagen type IV, fibronectin, and laminin are components of basement membrane. Protein GPIIb/IIIa and laminin belong to the same group of compounds, glycoproteins. Therefore, they are proper proteins to test biosensor specificity. Specificity of the interaction between antibody and laminin-5 was verified through treating a surface of the chip with immobilized antibody layer (5.0 ng mL^−1^) by mixtures of laminin-5 and potential interferents. Mixtures of different ratios of laminin-5 (0.05 ng mL^−1^) and interfering proteins were tested. The results are shown in Table 1. No influence of albumin, glycoprotein GPIIb/IIIa, fibronectin, and collage type IV on the results of determination of laminin-5 concentration was found. Even at 1000-fold excess of interferents used, SPRI sensors gave similar results. Thus, high selectivity of the biosensor was confirmed.

**Table 1:** Influence of human albumin, glycoprotein IIb/IIIa, fibronectin, and collagen on the determination of laminin-5 concentration by an SPRI method

Protein	*C*_laminin-5_/*C*_protein_	Added *C*_laminin-5_ (ng mL^−1^)	Found *C*_laminin-5_ (ng mL^−1^)	Recovery (%)
Albumin	1:1	0.050	0.050 ± 0.003	100
	1:100		0.052 ± 0.004	104
	1:1000		0.054 ± 0.003	108
Glycoprotein GPIIBb/IIIa	1:1	0.050	0.050 ± 0.005	100
	1:100		0.054 ± 0.002	108
	1:1000		0.055 ± 0.004	110
Fibronectin	1:1	0.050	0.050 ± 0.002	100
	1:100		0.054 ± 0.007	108
	1:1000		0.050 ± 0.002	100
Collagen type IV	1:1	0.050	0.051 ± 0.002	102
	1:100		0.055 ± 0.004	110
	1:1000		0.055 ± 0.004	110

### Precision and recovery of the method

Precision of the developed method was tested under optimal conditions, i.e., pH = 7.4 and antibody concentration of 5.0 ng mL^−1^. Experiments were performed for the solutions with laminin-5 concentrations of 0.1, 0.05, and 0.014 ng mL^−1^. Three plates with 12 free gold points were used for measuring each concentration. This gave 36 measuring points. The results are shown in Table 2.

**Table 2:** Precision of measurement of laminin-5 concentration

No. of measurements	Added *C*_laminin-5_ (ng mL^−1^)	Found *C*_laminin-5_ (ng mL^−1^)	Recovery (%)	SD	Confidence limit (95 %) (ng mL^−1^)
36	0.100	0.106	106	0.016	0.005
	0.050	0.052	104	0.012	0.004
	0.014	0.014	100	0.002	0.001

The precision of average value, as well as confidence limits, is acceptable. The recoveries of spikes are good, and they appear in the range of 100–106 %.

Recovery of the developed method was checked by the addition of standard solution of laminin-5 into nine different samples of the human plasma. The final standard concentration in the sample was 50.0 ng mL^−1^. The results are shown in Table 3. They are within the range of 98 to 106 %. The differences of determined concentrations were not statistically significant at *p* < 0.05.

**Table 3:** Recoveries of laminin-5 in the plasma

No. of samples	Laminin-5 concentration without spike (ng mL^−1^)	Added standard concentration (ng mL^−1^)	Determined (ng mL^−1^)	Recovery (%)
1	46.7 ± 1.2	50.0	94.8 ± 1.8	98
2	51.1 ± 2.0	50.0	107.2 ± 2.2	106
3	51.9 ± 1.5	50.0	101.9 ± 2.1	100
4	52.9 ± 1.9	50.0	101.9 ± 1.5	99
5	62.6 ± 1.1	50.0	110.3 ± 1.7	98
6	60.2 ± 1.7	50.0	111.3 ± 1.7	101
7	53.3 ± 1.5	50.0	105.4 ± 2.1	102
8	57.7 ± 1.5	50.0	113.1 ± 1.4	105
9	37.1 ± 2.1	50.0	91.5 ± 1.2	105

### Determination of laminin-5 concentration in biological samples

Samples of plasma from nine healthy adult donors and eight patients with bladder cancer were analyzed for laminin-5 concentration using the developed method. The results of measurements were evaluated on the basis of a calibration curve, and they are given in Table 4.

**Table 4:** Concentration of laminin-5 in the human blood plasma measured by SPRI method and ELISA

	Laminin-5 concentration (ng mL^−1^)			
	Healthy donors		Patients with bladder cancer	
	SPRI biosensor	ELISA	SPRI biosensor	ELISA
1	53.0 ± 1.5	57.9 ± 1.8	173.0 ± 2.5	138.0 ± 1.1
2	59.6 ± 1.1	61.1 ± 2.0	172.5 ± 3.1	109.8 ± 2.4
3	63.6 ± 0.9	63.7 ± 1.6	135.7 ± 1.6	121.2 ± 2.0
4	62.9 ± 0.7	15.5 ± 1.7	146.7 ± 2.8	109.1 ± 2.1
5	37.1 ± 1.2	40.9 ± 0.8	209.9 ± 1.9	151.7 ± 1.3
6	53.3 ± 0.8	52.2 ± 1.2	125.6 ± 1.9	91.0 ± 1.5
7	67.3 ± 0.9	65.8 ± 1.2	72.4 ± 2.7	63.5 ± 2.0
8	60.2 ± 0.6	61.1 ± 2.1	129.0 ± 3.0	112.6 ± 1.9
9	46.7 ± 1.5	44.4 ± 1.5		
Mean value ± confidence limit	56.0 ± 6.2	51.4 ± 10.4	145.6 ± 26.5	112.1 ± 17.6
Range	37.1–67.3	15.5–65.8	72.4–209.9	63.5–138.0
Literature range	46.2 ± 10.2 [20]			

In order to validate the developed method, comparative determination of laminin-5 concentration in the same samples by the commercial ELISA kit was performed (Table 4).

Generally, the comparison of results for separated samples from healthy donors obtained by SPRI measurement and ELISA indicates that they are close. For healthy subjects, the mean value of laminin-5 concentration was established as 56.0 ± 6.2 ng mL^−1^ with the SPRI biosensor and 51.4 ± 10.4 ng mL^−1^ with ELISA. The mean value of laminin concentration reported in the literature is 46.2 ± 10.2 ng mL^−1^ [20].

The laminin-5 concentration in the blood plasma of bladder cancer patients measured by both methods differed (mean values from the SPRI biosensor method was 145.6 ± 26.5 and 112.1 ± 17.6 ng mL^−1^ from ELISA). Also, all individual plasma samples of cancer patients gave significantly higher results measured with specific biosensor than by ELISA assay. It could be supposed that those differences came from disparate principles of both methods. ELISA assay was based on sandwich reaction of two different primary antibodies with two separated parts of the same molecule of the detected antigen. As a result, it needed longer polypeptide chain of investigated protein present in the biological sample than biosensor used for SPRI measurement. That biosensor was prepared by covering plate with one specific anti-laminin-5 antibody that could bind as short antigen molecule, as it contained specific epitope recognized by that used antibody. Blood plasma samples of cancer patients probably contained degradation products of laminin-5 with different lengths. About 23 % of them were so short that they were detected with a biosensor only. Even if those short fragments of laminin-5 were immobilized on ELISA plate, they were too small to bind second primary antibody of ELISA reagent. We tried to confirm that possibility by a Western immunoblot technique, as it was described earlier [32]. Unfortunately, there were no any bands on nitrocellulose reacting with the same primary anti-laminin-5 antibody that were used for biosensor preparation. It indicated that laminin-5 fragments detected with SPRI assay and ELISA test had lower molecular mass than about 7 kDa, which could be visualized by a Western blot technique.

At least three investigators found an increased laminin-5 loss from the basement membrane of the urinary bladder [18, 33] and its higher expression in bladder cancer [18, 34]. This might lead to laminin-5 higher turnover with a simultaneous increase in concentration of its degradation products in the blood of patients with urinary bladder cancer. Such significantly higher content was found especially with the proposed SPRI method. We supposed that the presented SPRI biosensor was an alternative to the standard methods for diagnosis of bladder cancer.

## Conclusions

The SPRI biosensors for the determination of laminin-5 concentration have been developed. The biosensor was based on the highly selective reaction between immobilized rabbit antibody and laminin-5. This method allows measuring in real-time interactions between biomolecules in a label-free manner with high precision and specificity of the assay.

Compared with the ELISA assay, the newly developed method proved to be a rapid, high-throughput, valuable, and reproducible tool for detection of laminin-5.

The newly developed SPRI biosensor was used successfully to determine laminin-5 concentration in serum. The study shows the clear difference in concentration of laminin-5 in healthy humans and patients with bladder cancer. Extensive clinical studies using the newly developed method can give rise to the use of laminin-5 as a potential cancer marker.

## Electronic supplementary material

Below is the link to the electronic supplementary material.

ESM 1 (PDF 3.57 mb)
