# Supplementary material for: SPR imaging biosensor for determination of laminin-5 as a potential cancer marker in biological material
Source: Anal Bioanal Chem. 2016 May 21;408:5269–76. doi: 10.1007/s00216-016-9621-x (PMC4925691; doi:10.1007/s00216-016-9621-x)

Analytical and Bioanalytical Chemistry

Electronic Supplementary Material

SPR imaging biosensor for determination of laminin-5 as a potential cancer marker in biological material

A. Sankiewicz, L. Romanowicz, P. Laudanski, B. Zelazowska-Rutkowska, B. Puzan, B. Cylwik, E. Gorodkiewicz

Fig.1. AFM pictures of bare gold (a), cysteamine (b), antibody (c), laminin-5 (d) on the chip.

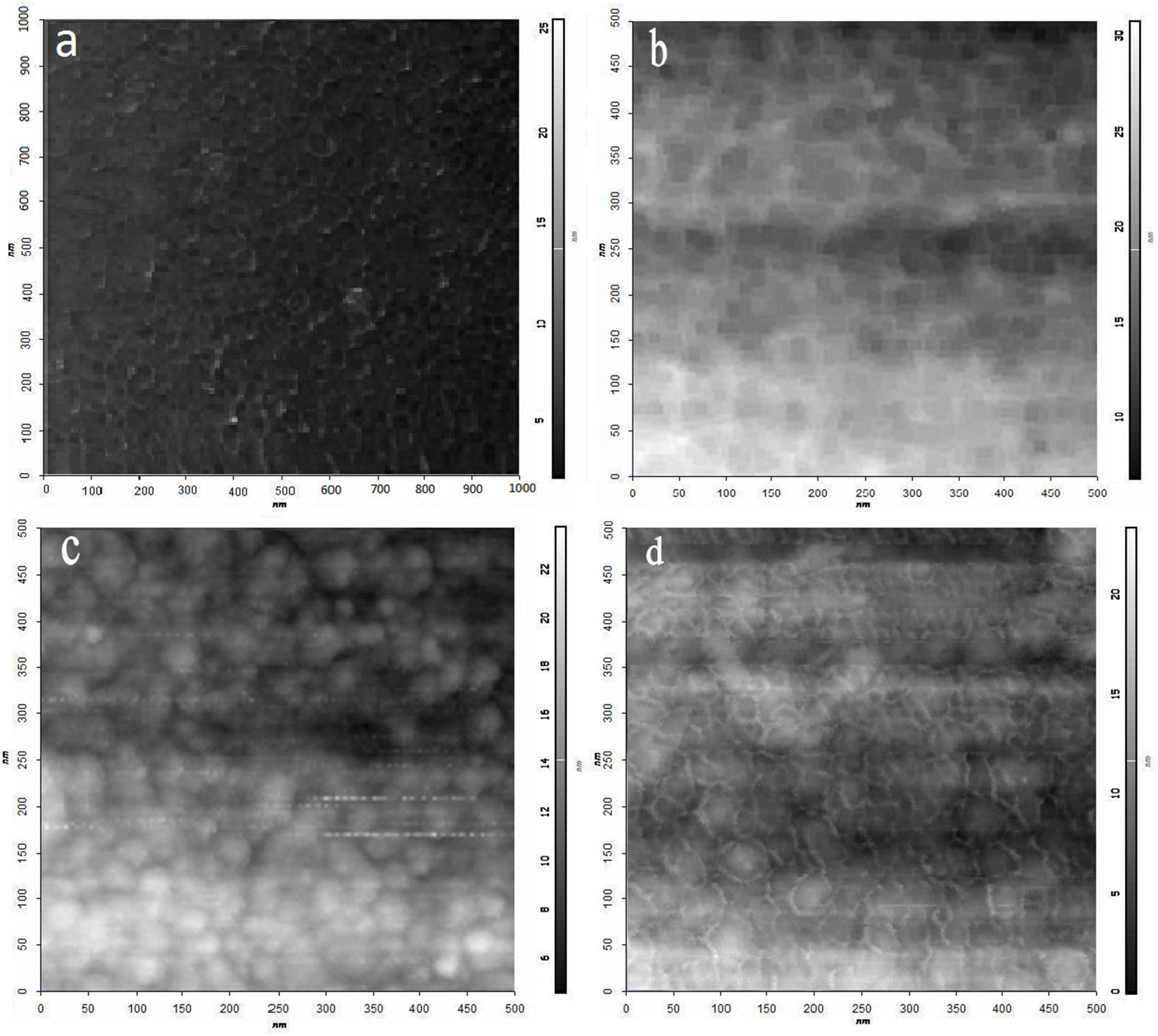

Supplement: Supplementary file 1 — (PDF 3.57 mb) [file 216_2016_9621_MOESM1_ESM.pdf]
